# N-acetylcysteine in Thrombotic Thrombocytopenic Purpura: A Systematic Review

**DOI:** 10.7759/cureus.109497

**Published:** 2026-05-23

**Authors:** Fawad Talat, Abdul Subhan Talpur, Maryam Sardar, Zeeshan Solangi, Hamza Usman, Areeba Ahmad, Amna Sardar, Mansi Kallem, Madhuri Yalamanchili

**Affiliations:** 1 Internal Medicine, King Edward Medical University, Lahore, PAK; 2 Internal Medicine, United Health Services (UHS) Wilson Medical Center, Johnson City, USA; 3 Medicine, Akhtar Saeed Medical and Dental College, Lahore, PAK; 4 Medicine, Yale University School of Medicine, New Haven, USA; 5 Medicine and Surgery, Continental Medical College, Lahore, PAK; 6 Hematology and Oncology, United Health Services (UHS) Wilson Medical Center, Johnson City, USA; 7 Medical Oncology, Broome Oncology, Johnson City, USA

**Keywords:** hematology, n-acetylcysteine, refractory ttp, therapeutics, thrombotic thrombocytopenia purpura

## Abstract

Background: Thrombotic thrombocytopenic purpura (TTP) is a life-threatening thrombotic microangiopathy characterized by severe ADAMTS13 deficiency and accumulation of ultralarge von Willebrand factor multimers. Although therapeutic plasma exchange (PEX), corticosteroids, rituximab, and caplacizumab have improved outcomes, refractory and relapsing disease remain important clinical challenges. N-acetylcysteine (NAC) has been explored as an adjunctive therapy because of its ability to reduce disulfide bonds within von Willebrand factor multimers.

Objective: This systematic review aimed to summarize the published clinical experience with NAC in TTP, including treatment strategies, reported outcomes, and safety.

Methods: A systematic search of PubMed was performed from database inception until December 31, 2025, using combinations of the terms “thrombotic thrombocytopenic purpura”, “TTP”, “N-acetylcysteine”, and “acetylcysteine”. Two reviewers independently screened records, assessed full texts against predefined eligibility criteria, and extracted study-level data. Eligible studies included case reports and case series describing therapeutic NAC use in patients with TTP. The review was conducted in accordance with PRISMA 2020 guidance. The protocol was not prospectively registered.

Results: Six publications comprising 22 adult patients were included. NAC was used as adjunctive therapy, predominantly in refractory or relapsing TTP, alongside PEX, corticosteroids, rituximab, and/or other immunosuppressive therapies. Platelet recovery was reported following NAC initiation in the included cases, with concurrent improvement in hemolysis markers and neurologic symptoms described in some reports. No serious NAC-related adverse events were reported in the included cases. Because all evidence was derived from uncontrolled reports with concomitant therapies, the independent contribution of NAC cannot be established.

Conclusions: The available case-based literature suggests that NAC is a biologically plausible and generally well-tolerated adjunctive therapy in refractory or relapsing TTP. However, the current evidence remains hypothesis-generating and is insufficient to establish efficacy. Prospective studies are needed to clarify its therapeutic role, optimal dosing, and patient selection.

## Introduction and background

Thrombotic thrombocytopenic purpura (TTP) is a rare but potentially fatal thrombotic microangiopathy characterized by thrombocytopenia, microangiopathic hemolytic anemia, and variable neurologic and organ dysfunction [1]. Acquired (immune-mediated) TTP results from autoantibodies against ADAMTS13, leading to impaired cleavage of ultralarge von Willebrand factor (UL-vWF) multimers and subsequent platelet-rich microvascular thrombosis [2,3].

Plasma exchange (PEX) combined with corticosteroids and immunosuppressive therapy has dramatically reduced mortality; however, refractory and relapsing disease remains a major clinical challenge [4,5]. The rationale for N-acetylcysteine (NAC) in TTP is supported primarily by mechanistic and preclinical evidence showing that NAC can reduce the size and activity of von Willebrand factor multimers by disrupting disulfide bonds, together with limited observational clinical reports. However, prospective controlled evidence remains lacking. Accordingly, NAC should be viewed as an investigational adjunctive strategy rather than an established therapy [6]. This mechanistic property has prompted off-label use of NAC in TTP. We conducted a systematic review of published case reports and case series evaluating NAC use in TTP following PRISMA guidelines [7].

Caplacizumab, an anti-von Willebrand factor nanobody, has become an important component of contemporary immune-mediated TTP management in combination with therapeutic PEX and immunosuppression. Guideline-based care increasingly incorporates caplacizumab during acute episodes because it improves early disease control and reduces exacerbations. Nevertheless, refractory and relapsing disease continue to occur, and access to targeted therapy may vary across settings [5].

## Review

Methods

Search Methodology

**Figure 1:**
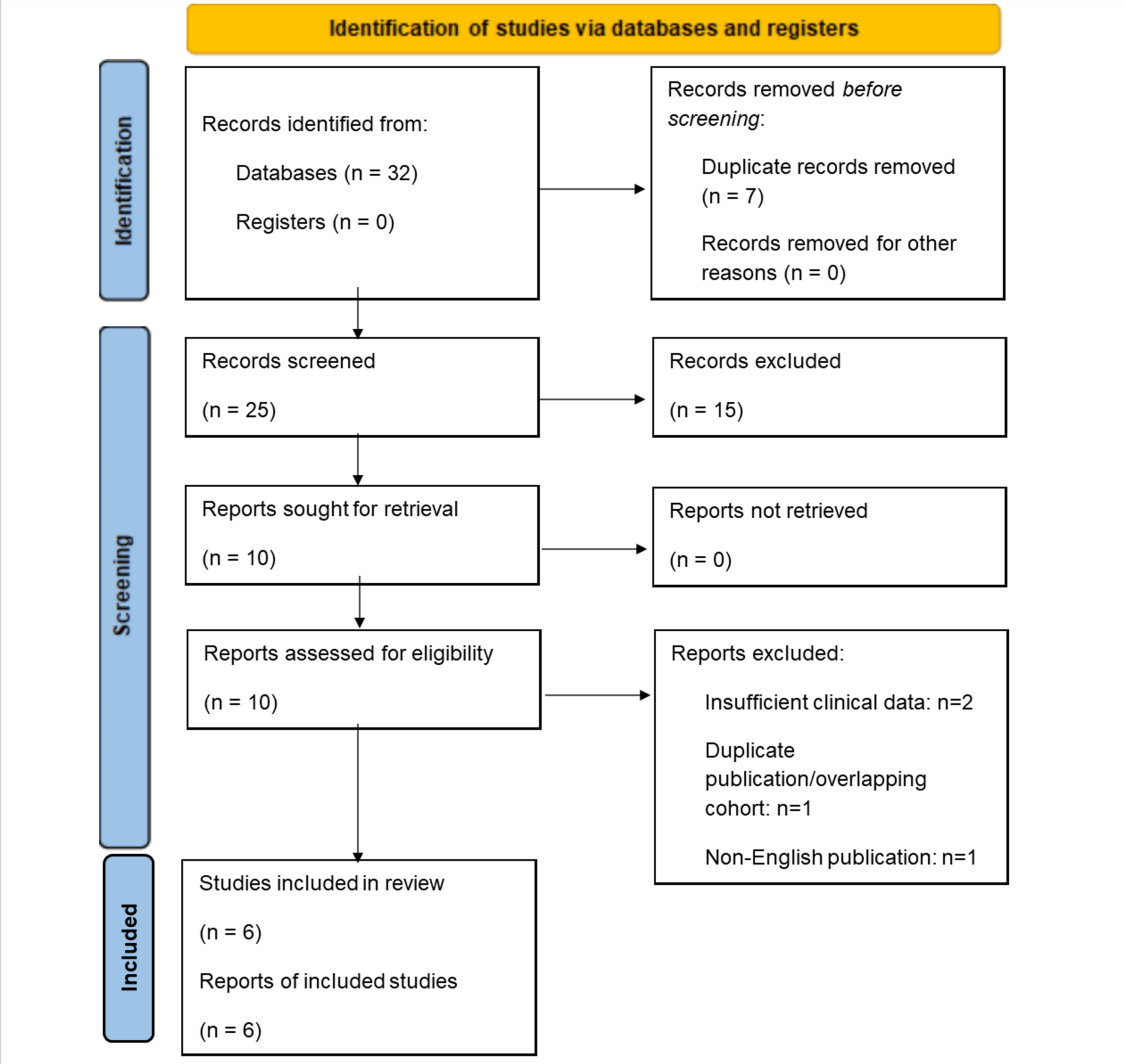
PRISMA 2020 flow diagram illustrating the study selection process for inclusion in this systematic review of N-acetylcysteine use in thrombotic thrombocytopenic purpura

A systematic literature search was conducted in PubMed from database inception until December 31, 2025. The following search strategy was used: (“thrombotic thrombocytopenic purpura” OR TTP) AND (“N-acetylcysteine” OR acetylcysteine OR NAC). No publication-date restrictions were applied. Only English-language publications were considered. Reference lists of relevant articles were also manually screened to identify additional eligible reports. Two reviewers independently screened titles and abstracts, reviewed full-text articles, and determined final eligibility according to predefined criteria. Disagreements were resolved by discussion and consensus (Figure 1).

Eligibility Criteria

The inclusion criteria were as follows: 1) human subjects; 2) patients diagnosed with TTP; 3) therapeutic administration of NAC; and 4) case reports, case series, or other eligible observational clinical reports providing patient-level or study-level clinical outcome data.

The exclusion criteria were as follows: animal studies, in vitro studies, narrative reviews without original clinical data, studies in which NAC use was unrelated to TTP management, and reports lacking sufficient clinical information for extraction.

For diagnostic classification, studies were categorized as confirmed immune-mediated TTP when severe ADAMTS13 deficiency and/or inhibitor positivity were explicitly documented. Reports without complete ADAMTS13 testing but with clinician-diagnosed TTP supported by thrombocytopenia, microangiopathic hemolytic anemia, and a compatible treatment course were classified as clinically suspected TTP.

Data Extraction and Synthesis

Data were extracted from all the included studies by two independent reviewers. The reviewers were tasked with extracting data on study design, number of patients, patient demographics, NAC dosing and duration, concomitant therapies, clinical outcomes, and adverse events. The results were synthesized and reported descriptively due to heterogeneity and a small sample size.

Risk of Bias

Methodological quality was assessed using the Joanna Briggs Institute (JBI) Critical Appraisal Checklists for case reports and case series, as appropriate to study design. Two reviewers independently performed the appraisal, and disagreements were resolved through discussion and consensus. Because the JBI tools are primarily domain-based rather than intended to generate a single validated numeric score, we summarized risk-of-bias concerns descriptively. Studies were considered to have lower concern when diagnostic characterization, intervention details, and outcome reporting were adequately described; moderate concern when one or more domains were incompletely reported; and higher concern when substantial uncertainty existed regarding diagnosis, treatment exposure, or outcome ascertainment (Table 1).

**Table 1:** Risk-of-bias assessment of included studies

Study	Study type	Selection bias	Diagnostic certainty	Intervention reporting	Outcome reporting	Overall risk of bias
Li et al. [8]	Case report	Moderate	Moderate	Adequate	Adequate	Moderate
Rottenstreich et al. [9]	Case series	Moderate	High	Adequate	Adequate	Moderate
Cabanillas and Popescu-Martinez [10]	Case report	Moderate	Moderate	Adequate	Adequate	Moderate
Demircioğlu et al. [11]	Case report	Moderate	Moderate	Limited detail	Adequate	Moderate
Español et al. [12]	Case series	Low	High	Detailed	Detailed	Low
Beyler and Demir [13]	Case series	Moderate	Moderate	Adequate	Adequate	Moderate

Each included study has been classified as low, moderate, or high risk of bias using this tool. A case series by Español et al. [12] demonstrated the lowest risk of bias, as patient characteristics, treatment protocols, and outcomes were more clearly defined.

Results

Study Selection

The PubMed search identified a limited body of literature describing the therapeutic use of NAC in TTP. After removal of nonrelevant articles, animal studies, and narrative reviews without original patient data, six publications met the inclusion criteria. No randomized trials or prospective cohort studies were identified. All included studies were published between 2014 and 2023.

Study and Patient Characteristics

Across the six included publications, a total of 22 adult patients with clinically diagnosed TTP received NAC. Patient ages ranged approximately from the third to the eighth decade of life, and both sexes were represented. All patients had typical features of TTP, including thrombocytopenia and microangiopathic hemolytic anemia. Some of the patients also had neurological manifestations, including confusion, visual disturbances, seizures, or focal deficits.

Most cases were categorized as immune‑mediated (acquired) TTP based on either documented severe ADAMTS13 deficiency with inhibitor positivity or a clinical presentation and treatment response consistent with acquired disease. However, not all reports included complete ADAMTS13 activity or inhibitor data, reflecting variability in diagnostic testing availability and reporting. No cases were explicitly identified as congenital TTP.

NAC was uniformly administered intravenously. The most common dosing used in these cases was 150 mg/kg/day. It was administered either as a continuous infusion or in divided doses. The total duration of treatment was 5-10 days. NAC was not used as monotherapy in all the included cases; rather, it was used as an adjunctive therapy.

Disease Setting and Concomitant Therapies

NAC was administered after the patients either failed to respond to several days of daily PEX and corticosteroids or had a relapsing disease with prior remission. NAC was predominantly started in patients with refractory or relapsing TTP. Refractory disease was typically defined as failure to achieve platelet recovery despite several days of daily PEX and corticosteroids. Relapsing disease occurred in patients with prior remission who experienced recurrent thrombocytopenia and hemolysis.

All patients received standard-of-care therapy, including therapeutic PEX and systemic corticosteroids. Most of the reported cases also received Rituximab, either prior to or along with NAC. In some cases, additional immunosuppressive agents were also used either before or concurrently with NAC.

Hematologic and Clinical Outcomes

Platelet recovery was reported following NAC initiation in the included cases. In some reports, platelet counts improved within days of NAC administration after a preceding period of limited response to conventional therapy. However, because NAC was administered together with or shortly after other treatments, including PEX, corticosteroids, rituximab, and additional immunosuppressive agents, the independent contribution of NAC cannot be determined.

Markers of hemolysis, including lactate dehydrogenase and indirect bilirubin, declined in parallel with platelet recovery in most patients. Some of the patients in the reported cases also exhibited neurological manifestations, and the neurological symptoms also improved after administration of NAC. There was a resolution of confusion, visual disturbances, and focal deficits.

There were no deaths attributable to TTP progression following NAC initiation. Despite favorable outcomes noted in the reports, the observational nature of the included studies limits definitive attribution of survival benefit.

Safety and Tolerability

No serious NAC-related adverse events were reported in the included cases. Minor infusion-related reactions were either absent or not described. However, safety conclusions remain limited by the small number of patients, observational reporting, and potential underreporting of adverse events.

Study Characteristics

The key design features, TTP classifications, NAC dosing strategies, and principal reported outcomes of the included studies are summarized in Table 2.

**Table 2:** Characteristics of included studies ^*^TTP type classification was based on the level of diagnostic confirmation reported in the included studies. Confirmed acquired TTP refers to cases with documented severe ADAMTS13 deficiency and/or inhibitor positivity, whereas suspected acquired TTP refers to clinically diagnosed cases without complete confirmatory ADAMTS13 or inhibitor testing TTP: thrombotic thrombocytopenic purpura; NAC: N-acetylcysteine

Study	Design	No. of patients	TTP type^*^	NAC dose	Key outcome
Li et al. [8]	Case report	1	Suspected acquired	150 mg/kg/day IV	Neurologic and platelet recovery
Rottenstreich et al. [9]	Case series	3	Acquired (ADAMTS13 confirmed)	High-dose IV	Platelet normalization in all
Cabanillas and Popescu-Martinez [10]	Case report	1	Suspected acquired	150 mg/kg/day IV ×10 days	Complete platelet recovery
Demircioğlu et al. [11]	Case report	1	Suspected acquired	High-dose IV	Clinical and hematologic improvement after NAC initiation
Español et al. [12]	Case series	12	Acquired (majority confirmed)	150 mg/kg/day IV	Median response 5.5 days
Beyler and Demir [13]	Case series	4	Acquired	High-dose IV	Clinical improvement in all

Patient and Treatment Characteristics

The overall patient demographics, disease setting, NAC administration details, treatment duration, and commonly used concomitant therapies are summarized in Table 3.

**Table 3:** Summary of patient-level clinical features TTP: thrombotic thrombocytopenic purpura; NAC: N-acetylcysteine; PEX: plasma exchange

Characteristic	Findings
Age	Adults (range ~30-75 years)
Sex	Both male and female
Disease setting	Refractory or relapsing TTP predominates
NAC route	Intravenous in all cases
NAC dose	Most commonly 150 mg/kg/day
Duration	5-10 days (typical)
Concomitant therapy	PEX, corticosteroids, rituximab ± other agents

Clinical Outcomes

The reported hematologic, neurologic, survival, and safety outcomes following NAC use are summarized in Table 4.

**Table 4:** Clinical outcomes and safety NAC: N-acetylcysteine

Outcome	Observation
Platelet recovery	Reported in majority of patients
Hemolysis markers	Improved concurrently
Neurologic recovery	Rapid improvement in severe cases
Survival	Favorable; no NAC-related deaths
Adverse events	No serious NAC-related toxicity reported

Discussion

This systematic review synthesizes all published cases describing the use of NAC as adjunctive therapy in patients with TTP. Although the available data are primarily derived from case reports and case series, the findings consistently demonstrate improvement in hematologic parameters and clinical outcomes following NAC administration, particularly in refractory or recurrent disease. These findings are noteworthy given the high morbidity that is associated with refractory TTP [14].

The biological rationale for NAC use in TTP is compelling. Severe ADAMTS13 deficiency leads to the accumulation of UL-vWF multimers, which promote platelet aggregation and microvascular thrombus formation [2,9]. While PEX removes circulating autoantibodies and replenishes ADAMTS13 activity, it does not directly neutralize circulating UL-vWF multimers [4]. NAC, through reduction of disulfide bonds within vWF multimers, decreases their size and platelet-binding activity independently of ADAMTS13 activity [6]. This mechanism may explain the improvement in platelet counts and neurological recovery observed in several of the reported cases included in this review.

In the included reports, NAC was typically introduced after patients failed to respond adequately to standard therapy, including PEX, corticosteroids, and rituximab. A temporal association was frequently observed between the initiation of NAC and subsequent platelet recovery, improvement in hemolysis markers, and resolution of neurologic symptoms. Although a causal relationship cannot be definitively established due to the observational nature of the available evidence and concurrent therapies, similar responses have been described in multiple independent case reports and case series [10-15].

A major interpretive limitation is the difficulty in isolating NAC’s independent therapeutic contribution. Across the included reports, NAC was generally introduced in patients already receiving PEX, corticosteroids, rituximab, and, in some cases, other immunosuppressive therapies. Therefore, although temporal associations between NAC initiation and subsequent hematologic or neurologic improvement have frequently been reported, these observations cannot establish causality. The current literature should be interpreted as hypothesis-generating rather than confirmatory.

Beyond case reports and case series, a retrospective cohort study of 89 adults with acquired TTP reported an association between NAC exposure and lower in-hospital mortality, although NAC use was not associated with faster platelet recovery or neurological recovery. As an observational study, these findings remain vulnerable to residual confounding and should not be interpreted as establishing a causal treatment effect [16].

It is important to acknowledge that ADAMTS13 activity levels and inhibitor testing were not uniformly reported across all included cases. Despite this limitation, the patients described were adults with clinical presentations and treatment courses typical of immune-mediated TTP, and no cases of congenital TTP were identified. This heterogeneity in diagnostic confirmation represents an important limitation and should be considered when interpreting the observed outcomes.

Across the reported cases, NAC was generally administered at high intravenous doses over several consecutive days. The therapy was well tolerated, and no serious adverse events directly attributable to NAC were reported. In addition to its favorable safety profile, NAC is widely available and relatively inexpensive. These characteristics may make NAC particularly appealing as an adjunctive therapy in refractory disease or in resource-limited settings where access to newer agents may be restricted.

The role of NAC should also be considered within the modern caplacizumab era. Caplacizumab is now integrated into contemporary management of acute immune-mediated TTP alongside PEX and immunosuppression. NAC may remain of interest as a possible adjunct in refractory or relapsing presentations, especially when disease remains difficult to control or where access to targeted therapies is limited. Nonetheless, current evidence is insufficient to define how NAC should be incorporated into contemporary treatment algorithms [5,15].

Despite the encouraging observations summarized in this review, several limitations must be acknowledged. The available evidence is derived entirely from case reports and case series, which are inherently subject to selection bias, small sample size, and potential publication bias. The descriptive nature of these studies also limits the ability to determine the independent contribution of NAC to clinical outcomes. Future prospective studies or registry-based analyses are needed to better define the optimal dosing, timing of administration, and patient populations most likely to benefit from NAC therapy. Additionally, further investigation is warranted to clarify how NAC might be integrated with emerging therapies such as caplacizumab in the management of TTP [5].

## Conclusions

Published case reports and case series suggest that NAC may be a safe and potentially effective adjunctive therapy in patients with refractory or relapsing TTP. NAC administration showed improvement in platelet count and a decrease in hemolysis marker, and was even associated with improvement in neurological symptoms. The observational nature of the included studies limits strong conclusions to be drawn from this review. The true magnitude of benefit attributable to NAC remains uncertain, as most patients received concomitant standard therapies. While current evidence is limited, the consistency of clinical responses and strong mechanistic rationale justify further prospective investigation. Randomized clinical trials can be designed to study the efficacy and safety of NAC therapy especially in patients with refractory/relapsing TTP.
